# The regulatory role of microRNA-1187 in TNF-α-mediated hepatocyte apoptosis in acute liver failure

**DOI:** 10.3892/ijmm.2012.888

**Published:** 2012-01-17

**Authors:** DONG SHAN YU, FANG MEI AN, BANG DONG GONG, XIAO GANG XIANG, LAN YI LIN, HUI WANG, QING XIE

**Affiliations:** 1 Department of Infectious Disease, Ruijin Hospital, Shanghai Jiaotong University School of Medicine, Shanghai; 2 Department of Infectious Disease, The Second Affiliated Hospital to Nanchang University, Nanchang, P.R. China

**Keywords:** acute liver failure, hepatocyte apoptosis, caspase-8, miR-1187, miR-1187 mimic

## Abstract

In the current study, we aimed at elucidating the regulatory mechanisms through which microR-1187 (miR-1187) participates in hepatocyte apoptosis in acute liver failure (ALF). An ALF model was induced with D-galactosamine (D-GalN) plus lipopolysaccharide (LPS) in BALB/c mice. The hepatic miRNA expression profile was detected by microarray analysis and verified by quantitative real-time PCR (qRT-PCR). The possible underlying mechanism was investigated *in vitro* using an embryonic murine hepatocyte cell line (BNLCL2) and miR-1187 mimic. Caspase-8 protein was detected by Western blotting and cell apoptosis was assayed by flow cytometry. Hepatic miR-1187 was down-regulated in ALF mice based on microarray data (P<0.001) and verified by qRT-PCR (P<0.01). Target scan revealed that caspase-8 was the putative target of miR-1187. In an *in vitro* study, miR-1187 showed the highest up-regulation in BNLCL2 cells transfected with the miR-1187 mimic at a 50 nM concentration for 12 h compared with cells transfected with the non-specific mimic (P<0.001). miR-1187 was down-regulated (P<0.01) but caspase-8 mRNA (P<0.01) as well as protein (P<0.05) were up-regulated in the BNLCL2 cells treated with D-GalN/TNF. Furthermore, overexpressed miR-1187 reduced caspase-8 expression at both the mRNA and protein levels significantly (P<0.01 and P<0.05 respectively), and significantly attenuated the apoptotic rate of BNLCL2 cells (P<0.05). We show that miR-1187 regulates hepatocyte apoptosis by targeting caspase-8. miR-1187 may serve as a potential therapeutic target for the treatment of ALF.

## Introduction

Acute liver failure (ALF) is a complex multisystemic disease with degeneration and necrosis of the liver which induces serious hepatosis, disturbances of blood coagulation, jaundice, hepatic encephalopathy and high mortality rates (1). Worldwide, the most frequent cause of ALF is viral hepatitis, such as hepatitis B and hepatitis C (2). Recent research suggests that apoptosis, infiltration of inflammatory cells and microcirculatory disturbance play important roles in the process of ALF (3,4). Apoptosis of hepatocytes was mediated by death receptors such as Fas, tumor necrosis factor (TNF)-α, and TNF related apoptosis-inducing ligand (TRAIL) (5,6), which have been implicated in hepatitis including hepatitis B virus (HBV) and hepatitis C virus (HCV) infection (7,8).

Caspase-8, a key protease in the death receptor signaling pathways, is activated when receiving apoplectic signals and is turned into cleaved caspase-8. Cleaved caspase-8 may activate caspase-3, caspase-9, eliminate Bcl-2 and may initiate apoptosis (9). Thus, caspase-8 is an ideal target factor for inhibiting apoptosis. The discovery of small non-coding RNA called microRNA (miRNA) has greatly expanded our understanding of the cellular mechanisms that regulate gene expression and immunology (10,11). Many studies have found that the miRNA expression profile is altered in liver diseases (12,13). Lanford *et al* reported that SPC3649 blocked miR-122 and effectively inhibited HCV replication and improved the pathological state of the liver in HCV model animals (14). Yoo *et al* unraveled a novel mechanism by which increased RNA-induced silencing complex (RISC) activity might contribute to hepatocarcinogenesis (15).

Our previous studies found that the expression profile of hepatic miRNAs in ALF mice is significantly altered. We found that hepatic miR-122, a liver specific miRNA, was decreased and correlated reversely with hepatic damage (16). We also found miRNAs including miR-155, miR-146a, miR-125a, miR-15b and miR-16 were up-regulated and miR-1187 was down-regulated significantly during ALF in mice (17). These studies suggest that miRNAs play regulatory roles in ALF. However, it is still unclear whether down-regulation of miR-1187 plays a role in hepatocyte apoptosis. In the current study, we report a possible role of miR-1187 in hepatocyte apoptosis in ALF mice.

## Materials and methods

### Animal model of ALF

Male BALB/c mice (10-weeks-old) weighing 20–22 g, obtained from Shanghai SLAC Laboratory Animal Co., Ltd., (Shanghai, China), were housed under conventional laboratory conditions with food and water *ad libitum*. Experiments adhered to the guidelines of the Shanghai Jiaotong University Animal Ethics Committee.

A murine ALF model was induced by intraperitoneal injection of D-GalN (Sigma-Aldrich, St. Louis, MO, USA) (900 μg/kg of body weight) and LPS (Sigma-Aldrich) (10 μg/kg body weight) as described (18), whereas the control group was given saline only. The challenged mice were sacrificed at different time points (n=8 per group). Part of the liver was stored in liquid nitrogen for qRT-PCR and Western blotting, whereas another part was fixed in 10% formalin for histopathology.

### Histopathology

Formalin fixed livers were embedded in paraffin, for routine histological analysis, 5 μm sections were cut and stained with hematoxylin and eosin (H&E).

### RNA extraction and quantitative real-time reverse transcription polymerase chain reaction (qRT-PCR)

Total RNA was extracted from liver tissue or cells, using Trizol (Invitrogen, Paisley, UK) according to the manufacturer’s instructions. qPCR was used to confirm the expression of miR-1187 and caspase-8 mRNA. cDNA was synthesized using reverse transcriptase (37°C for 30 min, 85°C for 5 min; Takara Bio, Inc., Ohtsu, Shiga, Japan). qRT-PCR was performed with the SYBR-Premix Ex TaqII (Takara Bio, Inc.) with the ABI 7500 qPCR system (95°C for 30 sec, 95°C for 5 sec, and 60°C for 34 sec) (Applied Biosystems, Foster City, CA, USA) (19). qPCR was performed, using primers described in Table I to detect mouse miR-1187 with U6 as an internal control, while for caspase-8 β-actin was used as internal control. The quantity of miRNA was calculated with the formula 2^−ΔΔCT^ (the C_T_ value represents fluorescence reached the threshold number of cycles required for PCR) (20).

### Cell culture and apoptosis induction

The normal mouse embryonic liver cell line (BNLCL2) (21) obtained from the Cell Bank, Chinese Academy of Science, was maintained in Dulbecco’s modified Eagle’s medium (DMEM; Invitrogen, Carlsbad, CA, USA) supplemented with 10% FBS (Gibco-Invitrogen, Carlsbad, CA, USA) and 1% streptomycin (Gibco-Invitrogen) at 37°C with 5% CO_2_. TNF-α (10 μg/ml; Sigma-Aldrich) and D-GalN (1.0 mg/ml; Sigma-Aldrich) were added into cell culture medium for induction of apoptosis (22), whereas the mock-treated cells were given culture medium only. Part of the cells was collected for total RNA extraction, while the other was collected for Annexin-V-FITC/PI-labeled (Mai Bio, Shanghai, China) flow cytometric analysis. FITC-positive and PI-negative cells were considered as apoptotic cells and PI-positive cells were considered as necrotic, while unstained cells were normal viable cells. FCSExpressV3Full software (De Novo Software, Thornhill, Canada) was requested for apoptotic profiles analysis.

### Transfection of the miR-1187 mimic and non-specific mimic

miRNA mimics are small double-stranded RNA oligonucleotides designed to mimic (overexpression) endogenous mature miRNA molecules when transfected into cells. The synthesized miR-1187 mimic and non-specific mimic (NSM) were purchased from RiboBio (Guangzhou, China). BNLCL2 cells (50–70% confluent) were transiently transfected with miR-1187 mimic (50 nM) using Lipofectamine 2000 (Invitrogen, Carlsbad, CA, USA) following the manufacturer’s instruction; NSM (50 nM) was transfected as a negative control. The efficiency of transfection was analysed using qRT-PCR.

### Western blot analysis

Total protein from BNLCL2 cells was extracted with extraction buffer RIPA plus the protease inhibitor PMSF (Bocai Bio Co., Shanghai, China) and quantified by the bicinchoninic acid (BCA) method (Pierce, Rockford, IL, USA). Protein samples were size-fractionated on 8% SDS-polyacrylamide gels and transferred to PVDF membranes (Amersham, Buckinghamshire, UK). The membranes were blocked with 5% dry milk for 1 h in Tris-saline buffer and 0.1% Tween-20 (TBS/Tween-20) (Dako, Carpinteria, CA, USA). After washing in TBS/Tween-20, the membranes were incubated with rabbit anti-mouse caspase-8 polyclonal antibody or rabbit anti-mouse β-actin polyclonal antibody (1:2,000, Abcam, Cambridge, UK) overnight at 4°C. After washing with TBS/Tween-20, the membranes were incubated for 1 h at room temperature with HRP-conjugated goat anti-rabbit IgG (1:2,000, Abcam, Cambridge, UK), washed with TBS/Tween-20 and the proteins of interest on the membrane were detected with ECL Plus™ Western blotting detection reagents (Amersham).

### Statistical analysis

All data are presented as means ± SD (standard deviation). Two-way ANOVA or Student’s t-test was performed for statistical analysis, using Graphpad Prism 5 (GraphPad Software, Inc.; La Jolla, CA, USA). Differences between group means with P<0.05 were regarded as being statistically significant.

## Results

### miR-1187 was significantly decreased during ALF and was predicted to target the caspase-8 mRNA 3′-untranslated region (3′UTR)

**Figure 1:**
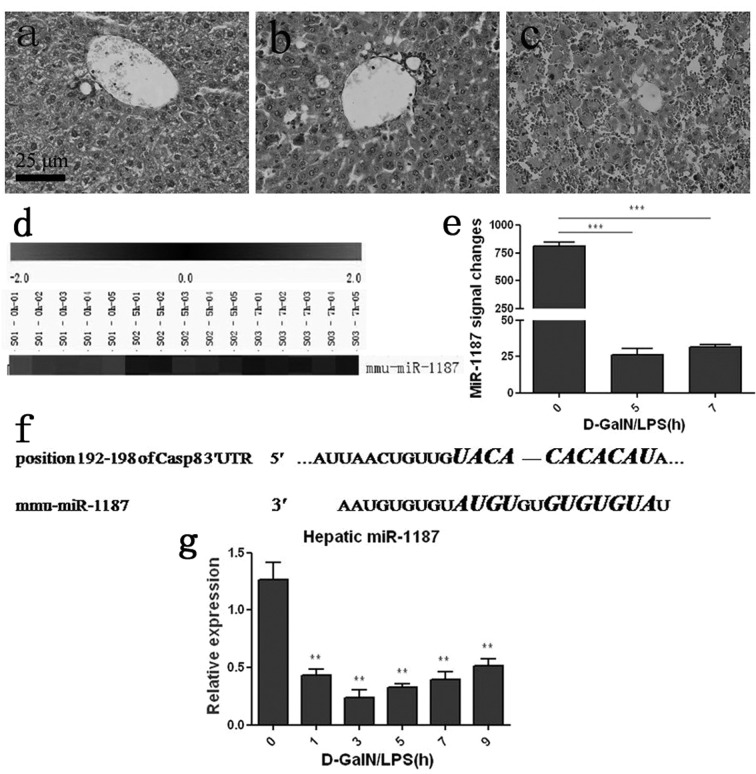
Histopathology and the expression of miR-1187 in the liver of ALF mice. Histological changes in livers at different time points post D-GalN/LPS induction (H&E, ×400). (a) There was no obvious abnormality in the liver at 0 h (saline control). (b) Damaged lobules of the liver were observed at 5 h. (c) More severe liver damage was observed at 7 h. (d) The expression of hepatic miR-1187 was then detected using the LAN-based microarray at 0, 5, 7 h post D-GalN/LPS treatment (n=5 for each time point). (e) Based on the microarray data, the change of the signal was quantified. (f) Predicted interactions between miR-1187 and the 3′UTR of caspase-8 mRNA by TargetScan. miR-1187 has binding sites at position 192–198 of caspase-8 mRNA 3′UTR highlighted in bold italic print. (g) qRT-PCR was used to verify the hepatic miR-1187 expression. Data represent the mean ± SD of five independent experiments. ^**^P<0.001, ^***^P<0.001.

After D-GalN/LPS induction, the mice mortality rate was 50% at 7 h and over 80% at 24 h (data was shown). A histopathology study detected obvious damage in the liver 5 h post-challenge, which showed a large quantity of inflammatory cells and disordered hepatic lobules (Fig. 1b). At 7 h, histopathological change was pronounced and severe congestion and destructed hepatic lobules were detected (Fig. 1c). LNA-based microarray analysis showed that hepatic miR-1187 was dramatically decreased with ongoing ALF (Fig. 1d). The miR-1187 expression signal was quantified and about a 97 or 96% decrease at 5 or 7 h post challenge respectively was noted compared with 0 h (saline control) (P<0.001, Fig. 1e). qRT-PCR was applied to verify the expression of miR-1187, and the result was consistent with the microarray data (P<0.01, Fig. 1g). TargetScan (http://www.targetscan.org/) revealed that 7 nucleotides in the seed region of miR-1187 were complementary to the position 192–198 of caspase-8 mRNA 3′UTR in mice (Fig. 1f).

### Overexpression of miR-1187 suppressed the level of caspase-8 mRNA as well as protein in BNLCL2 cells induced by D-GalN/TNF

**Figure 2:**
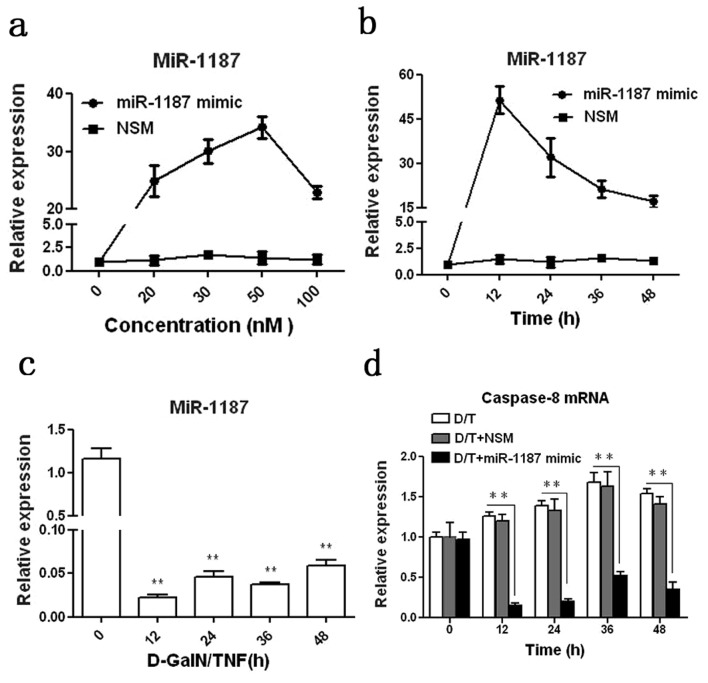
The expression of miR-1187 and caspase-8 mRNA in the BNLCL2 cells with different kinds of treatment. (a) miR-1187 expression in BNLCL2 cells transfected with a miR-1187 mimic or a non-specific mimic (NSM) at different concentration for 24 h. (b) miR-1187 expression in BNLCL2 cells transfected with miR-1187 mimic or NSM at the concentration of 50 nM for 48 h. (c) The expression of miR-1187 in the BNLCL2 cells induced by D-GalN/TNF. (d) The expression of caspase-8 mRNA in the BNLCL2 cells induced by D-GalN/TNF with or without 12 h miR-1187 (D/T+1187 mimic) or NSM (D/T + NSM) transfection. Data represent the mean ± SD of 3 independent experiments. ^**^P<0.01.

In order to study the regulatory role of miR-1187 in caspase-8, we applied a miR-1187 mimic for study. It was shown that miR-1187 was overexpressed in BNLCL2 cells transfected with miR-1187 mimic compared with the cells transfected with NSM. At 50 nM the miRA-1187 mimic with 12 h transfection resulted in the highest expression of miR-1187 in BNLCL2 cells (P<0.001, Fig. 2a and b). Thus we used this condition for the whole experiment. miR-1187 was dramatically decreased in the cells induced with D-GalN/TNF (P<0.01, Fig. 2c), correlating with our *in vivo* data above (Fig 1d, e and g). Inversely, caspase-8 mRNA was up-regulated in BNLCL2 cells induced with D-GalN/TNF (P<0.05), but overexpression of miR-1187 reduced caspase-8 mRNA significantly (P<0.01, Fig. 2d).

**Figure 3:**
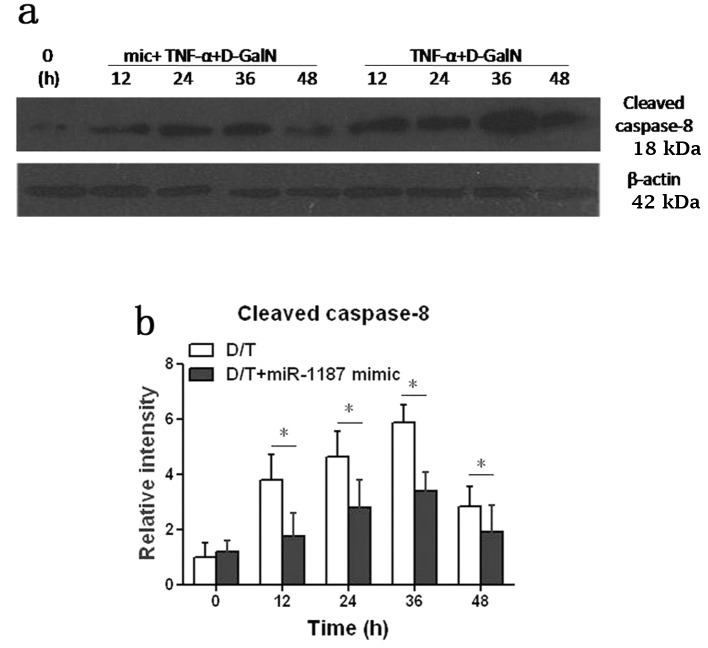
The regulatory role of miR-1187 in caspase-8 was detected by Western blot analysis. (a) The level of cleaved caspase-8 protein in BNLCL2 cells induced by D-GalN/TNF with or without miR-1187 mimic transfection (D/T+1187 mimic) was detected by Western blotting. (b) The signal of Western blotting was quantified using gray-scale analysis software and data was normalized to β-actin. Data represent the mean ± SD of 3 independent experiments. ^*^P<0.05.

Cleaved caspase-8 protein was increased in BNLCL2 cells induced by D-GalN/TNF, but it was significantly suppressed (P<0.05) when the cells were transfected with the miR-1187 mimic (D/T+1187 mimic) (Fig. 3). Taken together, it was presumed that miR-1187 regulated caspase-8 by mRNA degradation and the level of protein was suppressed accordingly.

### Up-regulated miR-1187 attenuated apoptosis of BNLCL2 cells

**Figure 4:**
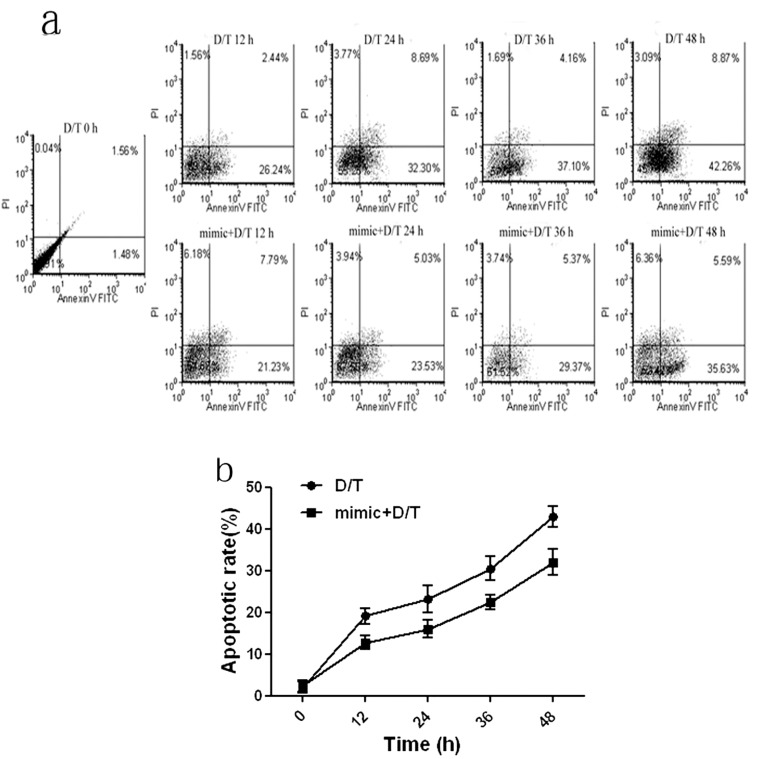
Apoptosis of BNLCL2 cells with different treatments were analyzed. (a) Apoptosis of BNCL2 cells was quantified by Annexin-V/PI labelled flow cytometric analysis in which Annexin-V-FITC was used to label apoptotic cells and PI was used to label necrotic cells in the treatment of the apoptosis of the cells treated with D-GalN/TNF for 12, 36 and 48 h (D/T 12, 24, 36, 48 h) or the cells transfected with miR-1187 mimic for 12 h then treated with D-GalN/TNF for another 12, 24, 36 and 48 h (mimic + D/T 12, 24, 36, 48 h) was detected. The mock treatment was used as a negative control. (b) Based on the flow cytometry data, the apoptosis rates were calculated. Data represent the mean ± SD of 8 independent experiments. ^*^P<0.05.

Following the data above, it is of interest to investigate if miR-1187 regulates BNLCL2 cells apoptosis. The flow cytometry data showed that the apoptotic rate of BNLCL2 cells was increased in a time-dependent manner; it was 26, 32, 37 or 42% following the D-GalN/TNF treatment for 12, 24, 36 or 48 h respectively. However, overexpression of miR-1187 attenuated the apoptotic rate significantly, i.e. by about 21, 24, 29 and 36%, respectively (P<0.05, Fig. 4).

## Discussion

Death receptor-mediated apoptosis of hepatocytes contributes to ALF (23). Recent studies found miRNAs are responsible for a number of liver diseases (24). Increasing evidence demonstrates that miRNAs regulate death receptor-mediated hepatocytes apoptosis in ALF (25,26). However, the regulatory role of miRNAs in TNF-α-dependent hepatocytes apoptosis in ALF remains unclear.

In the current study, the D-GalN/LPS induced ALF mice model was used for investigation. It was detected that miR-1187 was reduced in the liver of ALF mice by qRT-PCR. This data was consistent with our microarray data. Furthermore, using the TargetScan database, it was found that caspase-8 was a putative target of miR-1187. Fas, TNF-α and TRAIL are important genes in the death receptor-mediated apoptosis signaling pathway in human acute viral hepatitis (27,28), and caspase-8 is a key gene involved in this pathway. Caspase-8 has been implicated in hepatocyte apoptosis in liver injury (29), anti-caspase-8 could effectively inhibit apoptosis of Hepa 1-6 cells induced by TNF-α (30) and caspase-8 small interfering RNA prevents mice from acute liver failure (31). Thus, it is reasonable to conclude that miR-1187 contributes to hepatocyte apoptosis via targeting caspase-8 during ALF.

In order to study the role of miR-1187 in hepatocytes apoptosis, D-GalN/TNF was applied to induce BNLCL2 to apoptosis. After induction, a down-regulation of miR-1187 but an up-regulation of caspase-8 (both mRNA and protein level) was detected. Moreover, overexpression of miR-1187 reduced the levels of caspase-8 mRNA and protein and attenuated the apoptotic rate as well. Taken together, we speculate that overexpression of miR-1187 suppresses caspase-8, then down-regulates downstream genes including caspase-3, caspase-7 and caspase-9, consequently attenuating the apoptosis of hepatocytes. However, the relationship between miR-1187 and other factors related with immunization and apoptosis and how miR-1187 plays a role in an *in vivo* environment, remain to be further investigated.

In summary, our study demonstrated that miR-1187 regulated hepatocytes apoptosis via targeting caspase-8. miR-1187 acted as an inhibitor of hepatocyte apoptosis which shed light on the treatment of ALF.

## Figures and Tables

**Table I:** Primers for qRT-PCR and sequences of miR-1187 mimic and non-specific mimic.

Name	Sequence (5′-3′)
miR-1187	RT: GTCGTATCCAGTGCAGGGTCCGAGGTATTCGCACTGGATACGACTTACAC
	F: CCCGGCTATGTGTGTGTGTATGT
	R: GTGCAGGGTCCGAGGT
miR-1187 mimic	F: UAUGUGUGUGUGUAUGUGUGUAA
	R: AUACACACACACAUACACACAUU
Non-specific mimic	F: UCACAACCUCCUAGAAAGAGUAGA
	R: AGUGUUGGAGGAUCUUUCUCAUCU
U6	F: CTCGCTTCGGCAGCACA
	R: AACGCTTCACGAATTTGCGT
Caspase-8	F: TGCCCTCAAGTTCCTGTGCTTGGAC
	R: GGATGCTAAGAATGTCATCTCC
β-actin	F: CTAGGCACCAGGGTGTGAT
	R: TGCCAGATCTTCTCCATGTC

RT, miRNA special reverse transcription primer; F, forward primer; R, reverse primer.
